# Dietary Mannan Oligosaccharides Enhance the Non-Specific Immunity, Intestinal Health, and Resistance Capacity of Juvenile Blunt Snout Bream (*Megalobrama amblycephala*) Against *Aeromonas hydrophila*

**DOI:** 10.3389/fimmu.2022.863657

**Published:** 2022-06-15

**Authors:** Zhujin Ding, Xu Wang, Yunlong Liu, Yancui Zheng, Hongping Li, Minying Zhang, Yang He, Hanliang Cheng, Jianhe Xu, Xiangning Chen, Xiaoheng Zhao

**Affiliations:** ^1^ Jiangsu Key Laboratory of Marine Bioresources and Environment, Co-Innovation Center of Jiangsu Marine Bio-industry Technology, Jiangsu Ocean University, Lianyungang, China; ^2^ School of Marine Science and Fisheries, Jiangsu Key Laboratory of Marine Biotechnology, Jiangsu Ocean University, Lianyungang, China; ^3^ Key Laboratory of Sichuan Province for Fishes Conservation and Utilization in the Upper Reaches of the Yangtze River, Neijiang Normal University, Neijiang, China

**Keywords:** mannan oligosaccharides, *Megalobrama amblycephala*, non-specific immunity, intestinal health, immunoprotective effects

## Abstract

Mannan oligosaccharides (MOS) have been studied and applied as a feed additive, whereas their regulation on the growth performance and immunity of aquatic animals lacks consensus. Furthermore, their immunoprotective effects on the freshwater fish *Megalobrama amblycephala* have not been sufficiently studied. Thus, we investigated the effects of dietary MOS of 0, 200, and 400 mg/kg on the growth performance, non-specific immunity, intestinal health, and resistance to *Aeromonas hydrophila* infection in juvenile *M. amblycephala*. The results showed that the weight gain rate of juvenile *M. amblycephala* was not significantly different after 8 weeks of feeding, whereas the feed conversion ratio decreased in the MOS group of 400 mg/kg. Moreover, dietary MOS increased the survival rate of juvenile *M. amblycephala* upon infection, which may be attributed to enhanced host immunity. For instance, dietary MOS increase host bactericidal and antioxidative abilities by regulating the activities of hepatic antimicrobial and antioxidant enzymes. In addition, MOS supplementation increased the number of intestinal goblet cells, and the intestine was protected from necrosis of the intestinal folds and disruption of the microvilli and junctional complexes, thus maintaining the stability of the intestinal epithelial barrier. The expression levels of *M. amblycephala* immune and tight junction-related genes increased after feeding dietary MOS for 8 weeks. However, the upregulated expression of immune and tight junction-related genes in the MOS supplemental groups was not as notable as that in the control group postinfection. Therefore, MOS supplementation might suppress the damage caused by excessive intestinal inflammation. Furthermore, dietary MOS affected the richness and composition of the gut microbiota, which improved the gut health of juvenile *M. amblycephala* by increasing the relative abundance of beneficial gut microbiota. Briefly, dietary MOS exhibited significant immune protective effects to juvenile *M. amblycephala*, which is a functional feed additive and immunostimulant.

**Graphical Abstract:**
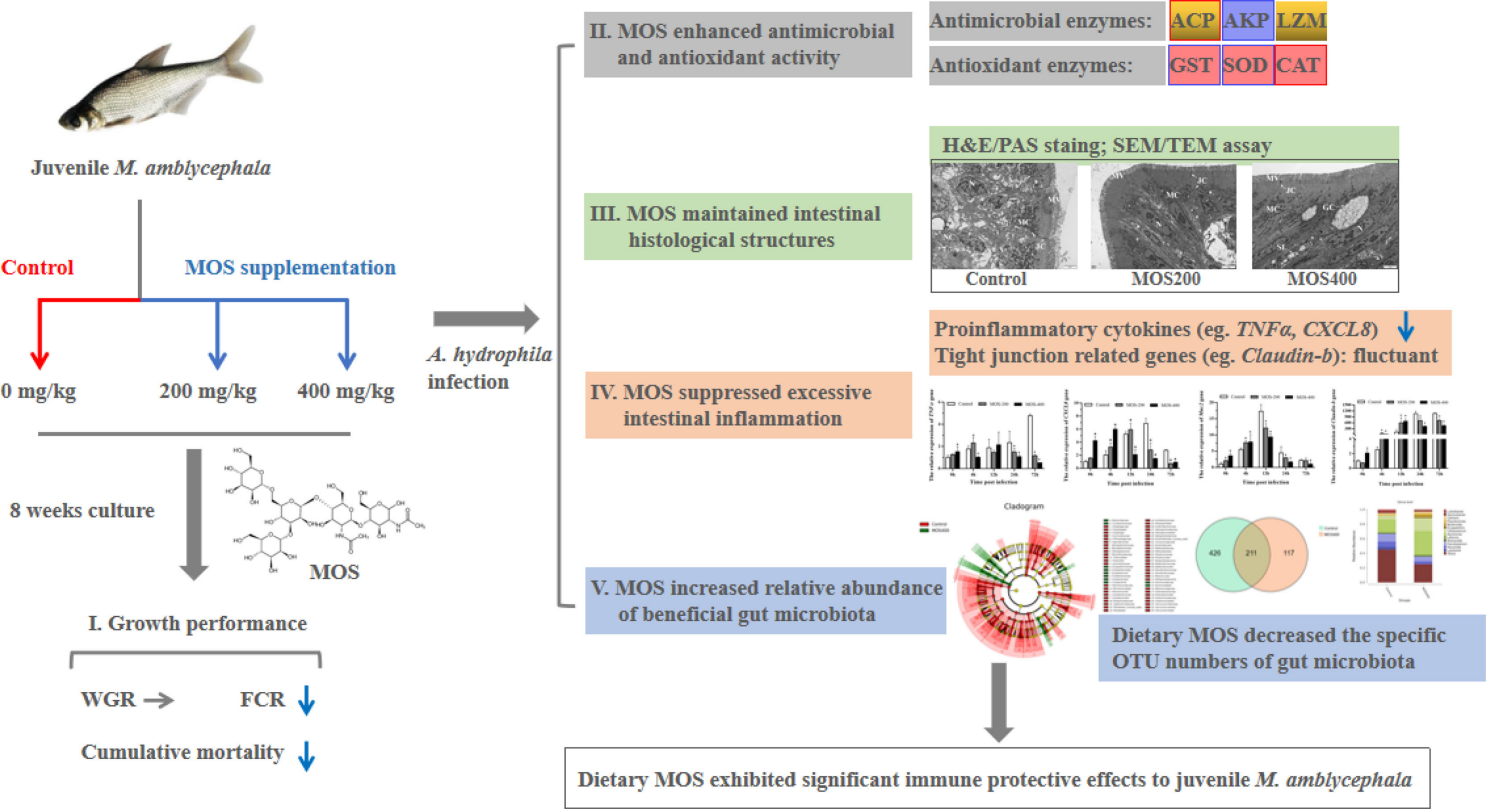


## Introduction

According to their biological functions, oligosaccharides may be divided into nutritional oligosaccharides, which are digested and absorbed to provide energy, and functional oligosaccharides, which are not easily digested but have special biological functions. Compared to probiotics, oligosaccharides can reach the intestinal tract and avoid inactivation. Thus, functional oligosaccharides are considered ideal feed additives; these include chitooligosaccharides, mannan oligosaccharides (MOS), fructooligosaccharides, soy oligosaccharides, xylooligosaccharides, and isomaltose (1, 2).

MOS have been widely studied and used as a feed additive in livestock and poultry cultures, but their application in aquaculture is relatively rare (3, 4). Several studies have revealed the effects of MOS on growth performance and the feed conversion ratio of aquatic animals (5); however, the results are not consistent, which might be related to factors such as MOS supplemental levels, culture environment, dietary nutrient levels, animal species, and the developmental stage (5, 6). In addition, studies on crucian carp (7), *Labeo rohita* (8), and other fish have shown that MOS supplementation can improve the survival rate of juvenile fish upon bacterial infection. The protective mechanisms of MOS might include improving the gut microbiota, reducing the colonization of pathogens, and enhancing host antioxidative ability (5). For instance, MOS can activate the MR/PKCδ signaling pathway in *Ctenopharyngodon idella*, thereby improving intestinal antioxidative ability (9).

The mechanical barrier, composed of intestinal epithelial cells, is the most important barrier in the intestinal mucosa. MOS play a protective role in the intestinal epithelial barrier by regulating the expression of tight junction proteins in chickens, rats, and pigs. For example, MOS reduce intestinal mucosal barrier damage in rats with acute pancreatitis by increasing the expression of *claudin-1*, *ZO-1*, and *mucin 2* (*muc2*) (10). In addition, MOS have also been shown to protect the intestinal epithelial barrier of pigs by upregulating the expression of *ZO-1* and *claudin-1* following *Escherichia coli* infection (11). Furthermore, the immunoprotective effects of MOS are related to its protection of the integrity of the intestinal mucosal barrier, including the enhancement of the tight junction structures between intestinal epithelial cells and maintenance of the length and density of the microvilli (6, 12). An established *in vitro* model using the *Oncorhynchus mykiss* intestinal epithelial cell line (RTgutGC) showed that MOS exhibit better protective effects on intestinal immunity and barrier functions than nucleotides and β-glucan (13).

However, the protective effects and mechanisms of MOS upon intestinal infection in *Megalobrama amblycephala*, one of the major freshwater fish in China, have not been sufficiently studied. Recently, because of the degradation of germplasm resources and environmental pollution, diseases have occurred frequently during *M. amblycephala* culture, among which the most serious is bacterial septicemia caused by infection with *Aeromonas hydrophila*. The infectious processes of *A. hydrophila* occur mainly through the intestinal tract, which penetrates the intestinal mucosal barrier and then proliferates and infects other parts of the host (14). Therefore, under the current background of antibiotic reduction and substitution, it is of great significance to develop antibiotic substitutes that can maintain intestinal health and intestinal mucosal barrier stability for the healthy culture of *M. amblycephala*.

Considering the immune regulatory functions of MOS, including enhancement of non-specific immunity, anti-infection ability, and the intestinal health of cultured animals, this study aimed to explore the protective effects of MOS in alleviating intestinal barrier injury in juvenile *M. amblycephala* upon bacterial infection. This study provides new ideas for regulating fish intestinal immunity and a theoretical basis for developing new immune agents and antibiotic substitutes.

## Materials and Methods

### Ethics Statement

This study was approved by the Animal Care and Use Committee of Jiangsu Ocean University (protocol no. 2020-37; approval date: September 1, 2019). All animal procedures were performed in accordance with the Guidelines for the Care and Use of Laboratory Animals in China.

### Dietary Formulation

On the basis of the nutritional requirements of *M. amblycephala*, an isonitrogenous and isoenergy basal diet was prepared with fish, soybean, cottonseed, and rapeseed meal as protein sources, soybean oil as a lipid source, and wheat middling as a carbohydrate source. The experimental diets of the MOS200 and MOS400 groups were formulated by supplementing MOS of 200 and 400 mg/kg (Alltech, Beijing, China) in the basal diet, respectively, and the components are shown in **Table 1**. First, all powdered ingredients were weighed and mixed for 10 min, and distilled water was added to the premixed dry ingredients and mixed for 15 min. Then, a proper pelletizing aperture (approximately 1.5 mm) was set according to the size of the experimental fish, and the diets were broken up into granules and dried in a drying oven to ensure a moisture content below 10%.

**Table 1:** Ingredients and nutrient composition of the experimental diets (%).

Ingredients	Groups		
	Control	MOS200	MOS400
Fish meal	8.00	8.00	8.00
Soybean meal	20.80	20.80	8.00
Cottonseed meal	15.00	15.00	15.00
Rapeseed meal	18.00	18.00	15.00
Wheat middling	30.00	30.00	30.00
Soybean oil	5.00	5.00	5.00
Ca(H_2_PO_4_)_2_	2.00	1.98	1.96
MOS	–	0.02	0.04
Choline	0.30	0.30	0.30
Vitamin premix^1)^	0.40	0.40	0.40
Mineral premix^2)^	0.50	0.50	0.50
Total	100	100	100
Nutrient levels^3)^			
Moisture	6.88	6.89	6.91
Crude protein	37.10	36.78	36.91
Crude lipid	8.43	8.41	8.28
Ash	7.50	7.51	7.51

^1)^ Vitamin premix for each kilogram of feed: VE, 50 mg; VA, 5,000 IU; VB1, 8 mg; VK, 5 mg; VB6, 8 mg; VD, 2,000 IU; VB2, 10 mg; pantothenic acid, 30 mg; VB12, 0.03 mg; folic acid, 3 mg; niacin, 30 mg; inositol, 100 mg; biotin, 0.4 mg; VC, 180 mg.

^2)^ Mineral premix for each kilogram of feed: Mg, 300 mg; Zn, 150 mg; Fe, 170 mg; Co, 0.25 mg; Cu, 4 mg; Mn, 22 mg; Se, 0.4 mg.

^3)^ Calculated values.

### Fish Rearing and Growth Performance Analysis

Juvenile *M. amblycephala*, obtained from a fish farm in Guangzhou, China, were fed with commercial feed for temporary rearing and taming for 2 weeks before the culture experiment. Fish husbandry was conducted in an indoor freshwater recirculating system consisting of 18 fiberglass tanks (90 L per tank) with equal supplemental aeration and water flow (1 L/min). In total, 495 experimental fish with a body weight of 0.87 ± 0.05 g were randomly assigned into three groups, including the control, MOS200, and MOS400 groups, and each group had three replicates (55 fish per tank). The experimental fish were cultured for 8 weeks and fed four times daily (8:00, 11:00, 14:00, and 17:00) to apparent satiation (approximately 3% of the body weight), and the water was renewed every day to maintain acceptable water quality. The water temperature was maintained at 26°C–28°C; the pH was approximately 7.2; ammonia, nitrogen, and nitrite were lower than 0.1 mg/L; and dissolved oxygen was greater than 6.0 mg/L.

The initial and final body weight and total feed intake were measured before and after the rearing experiment, respectively. The relevant growth index calculation formulas are specified below.


Weight gain rate (WGR) = 100% ×(final body weight - initial body weight)/initial body weight

Feed conversion ratio (FCR) =total feed intake (g)/weight gain (g)

### Bacterial Challenge and Sample Collection

The bacterial challenge was performed after 8 weeks of culture as previously described (15), and experimental fish from the control, MOS200, and MOS400 groups (55 fish per tank) were assigned to two categories for calculating mortality (20 fish per tank) and sample collection (35 fish per tank), respectively. Then, the experimental juvenile fish with a body weight of 4.21 ± 0.19 g were injected intraperitoneally with 0.1 ml (1 × 10^6^ CFU/ml) of *A. hydrophila* (LD50 dose). Three individuals from each tank were randomly dissected after anesthetized with 3-aminobenzoic acid ethyl ester methane sulfonate (MS-222; Merck KGaA, Darmstadt, Germany), and the hepatopancreas and intestines were removed at 0, 4, 12, 24, and 72 h postinfection (hpi). The hepatopancreas was homogenized for enzyme activity analysis, and the intestines were collected for histological assay, gut microbiota sequencing, and gene expression analysis.

### Analyses of Antimicrobial and Antioxidant Enzymes Activities

The excised hepatopancreas was weighed, and according to a ratio of tissue weight (g) to phosphate-buffered saline volume (ml) of 1:9, the hepatopancreas samples were homogenized using a high-throughput tissue crushing instrument. After centrifugation at 2,500 rpm for 10 min, the supernatant was extracted to determine the activities of hepatic antimicrobial and antioxidant enzymes. The activities of acid phosphatase (ACP), alkaline phosphatase (AKP), catalase (CAT), superoxide dismutase (SOD), glutathione S-transferase (GST), and lysozyme (LZM) were determined using the a corresponding enzyme activity detection kit (Nanjing Jiancheng Bioengineering Institute, Nanjing, China) according to the manufacturer’s instructions.

### Histological Assay

Hematoxylin and eosin (H&E) and Alcian blue (AB)–periodic acid–Schiff (PAS) staining of *M. amblycephala* intestinal sections were conducted to detect histological structures and goblet cell distribution, as previously described (16, 17). Briefly, fresh mid-intestinal tissues were fixed in 4% paraformaldehyde for 24 h at 4°C. Then, they were dehydrated with gradient ethanol, cleaned in xylene substitute, embedded in paraffin blocks, and sectioned at 4 μm thickness on a microtome. Subsequently, they were floated in a 40°C water bath, adhered onto glass slides, and dried in an oven at 40°C overnight. After deparaffinization and rehydration, the slides were stained with H&E or AB-PAS (Sigma, St. Louis, MO, USA). The sections were examined and photographed using a light microscope (Nikon, Tokyo, Japan). Then, a transmission electron microscopy (TEM) assay of the intestinal samples was performed, as previously described (12). The TEM micrographs (magnification, ×30,000) were obtained to measure the length and integrity of the microvilli and the pathological symptoms postinfection. All images were analyzed using Image-Pro Plus 6.0 (National Institutes of Health, Bethesda, MD, USA) to calculate the villus length, crypt depth, microvillus length, and the number of goblet cells.

### Total RNA Isolation and cDNA Preparation

Total RNA was extracted from the intestinal samples using the RNA Easy Fast Tissue Kit (TIANGEN, Beijing, China), according to the manufacturer’s instructions. The quality and concentration of total RNA were determined by agarose gel electrophoresis and NanoDrop 2000 (Thermo Fisher Scientific, Wilmington, DE, USA), respectively. In addition, cDNA was synthesized using the PrimeScript^®^ RT reagent Kit with gDNA Eraser (TaKaRa, Dalian, China) following the manufacturer’s protocol and stored at −20°C for real-time quantitative reverse transcription polymerase chain reaction (qRT-PCR).

### Real-Time qRT-PCR Analysis

The expression patterns of tight junctions and immune-related genes were analyzed using qRT-PCR, as previously reported (18). Briefly, qRT-PCR was performed with an ABI StepOne Plus real-time PCR system (PerkinElmer Applied Biosystems, CA, USA) using the QuantiNova™ SYBR^®^ Green PCR Kit (TaKaRa, Dalian, China) according to the manufacturer’s protocol. Relative expression levels of the target genes were measured in terms of the threshold cycle (Ct) value using the 2^−ΔΔCt^ method (19), and glyceraldehyde-3-phosphate dehydrogenase (*GAPDH*) was selected as the internal reference according to the geNorm software analysis (20). All the reactions were performed in triplicate, and the primers are listed in **Supplemental Table 1**. The gene expression levels in the control group were set as 1, and the relative expression levels of the MOS supplemental groups were presented as fold change.

### High-Throughput Sequencing of Intestinal Microorganisms

The total intestinal microbial genomic DNA was extracted for 16S rDNA high-throughput sequencing. The primer sequences for V3–V4 region amplification were as follows: F: 5′-NNNNNNNNACTCCTACGGGAGGCAGCAG-3′ and R: 5′-GGACTACHVGGGTWTCTAAT-3′. The melting temperature was 56°C, and amplification was conducted for 30 cycles. The validated libraries were paired-end sequenced using an Illumina HiSeq system (HiSeq reagent kit; Illumina, San Diego, CA, USA). The raw data were preprocessed by filtering out adapter contamination and low-quality sequences to obtain clean reads. Paired-end clean reads with overlaps were assembled into tags using Fast Length Adjustment of Short reads (FLASH) software (v1.2.11; 21). Then, de-noised bacterial tags were clustered to generate operational taxonomic units (OTUs) with 100% sequence similarity using the Divisive Amplicon Denoising Algorithm (DADA2) in the software QIIMA2 (22).

Bacterial OTU representative sequences were taxonomically classified using Mothur (v1.31.2) software based on the Ribosomal Database Project (23). Chao 1, Shannon, and Simpson indices were also calculated using Mothur, and rarefaction curves were drawn using R (v3.0.3) software. A beta-diversity analysis based on weighted UniFrac distance was conducted using QIIME (v1.80) software and visualized using principal coordinate analysis (PCoA) and heatmaps. In addition, a linear discriminant analysis (LDA) effect size (LEfSe) analysis was conducted using the LEfSe software (24).

### Statistical Analysis

In the present study, data are presented as the mean ± standard error (SE). Statistical significance was assessed using a one-way analysis of variance (ANOVA), and multiple comparisons were performed using the Tukey method in SPSS 25.0. Statistical significance was set at *P* < 0.05.

## Results

### Dietary MOS Improve the Feed Utilization Efficiency of Juvenile *M. amblycephala*

The effects of MOS supplementation on growth performance and the feed conversion ratio of juvenile *M. amblycephala* are presented in **Table 2**. There was no significant difference in the final weight and weight gain rate between the control and MOS supplemental groups, but the feed conversion ratio of the MOS400 group was significantly lower than that of the control group, indicating that dietary MOS could improve the feed utilization efficiency of juvenile *M. amblycephala*.

**Table 2:** Effect of dietary MOS on the growth performance and feed conversion ratio of juvenile *M. amblycephala* (mean ± SE).

Items	Groups		
	Control	MOS200	MOS400
Initial weight (g)	0.86 ± 0.05	0.87 ± 0.04	0.88 ± 0.06
Final weight (g)	4.12 ± 0.10	4.31 ± 0.12	4.12 ± 0.31
Weight gain rate (%)	369.04 ± 34.70	389.96 ± 44.40	358.36 ± 58.69
Feed conversion ratio	1.65 ± 0.05^a^	1.61 ± 0.08^a^	1.50 ± 0.05^b^
Survival rate (%)	100.00 ± 0.00	99.04 ± 1.64	98.09 ± 1.34

In the same row, values with no letter or the same superscript letter meant no significant difference (P > 0.05), whereas values with different superscript letters meant significant differences (P < 0.05).

### Dietary MOS Increase the Survival Rate of Juvenile *M. amblycephala* Postinfection

**Figure 1:**
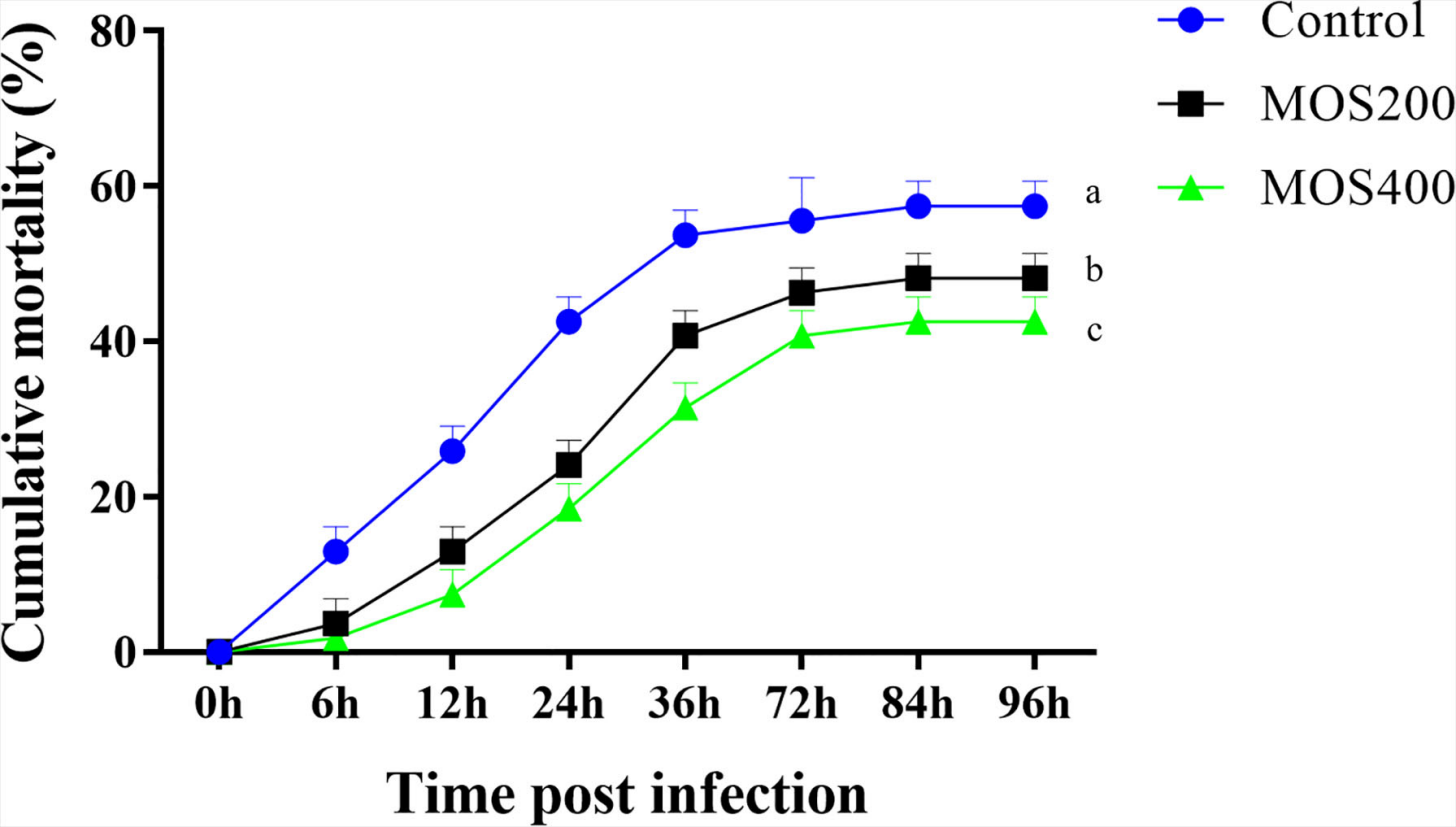
Dietary MOS decreased the mortality of juvenile *M. amblycephala* post–bacterial infection. Different letters indicated significant differences among groups (P < 0.05).

As shown in **Figure 1**, the cumulative mortality of all groups increased markedly from 6 hpi and reached the maximum at 84 hpi. In addition, the mortality rate of the control group was significantly higher than that of the MOS400 and MOS200 (200 mg/kg) groups at all time points. The final cumulative mortalities of the control, MOS200, and MOS400 groups were 55.6%, 46.3%, and 44.4%, respectively, indicating that MOS supplementation had a significant immunoprotective effect on juvenile *M. amblycephala.*

### MOS Enhance the Activities of Hepatic Antimicrobial and Antioxidant Enzymes

**Figure 2:**
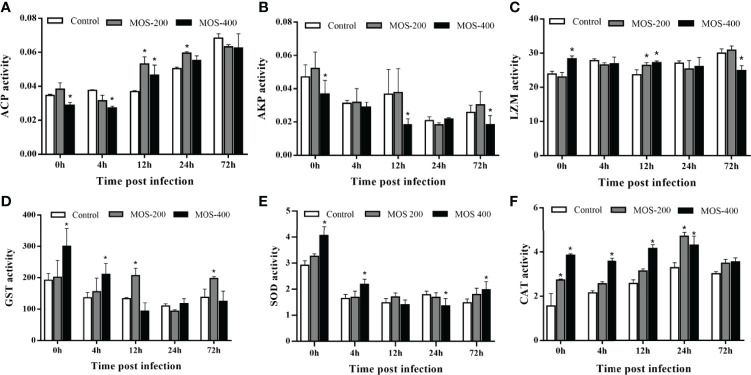
Effects of MOS supplementation on the activities of hepatic antimicrobial and antioxidant enzymes of juvenile *M. amblycephala*. **(A–F)** showed the enzymes activity of ACP, AKP, LZM, GST, SOD, and CAT, respectively. The asterisks indicated statistically significant differences among different groups at a certain time point (P < 0.05).

To assess the effects of MOS supplementation on the antimicrobial and antioxidant functions of juvenile *M. amblycephala*, we detected the activities of a series of hepatic enzymes (**Figure 2**). The ACP activity increased significantly upon infection with *A. hydrophila* in all groups, and that of the MOS400 group was much lower, at 0 and 4 hpi to maintain host homeostasis, but was notably induced in the MOS supplemental groups at 12 and 24 hpi to enhance the bactericidal effects. In addition, LZM activity was upregulated in the control and MOS200 groups postinfection, and that of the MOS400 group maintained relatively high levels at all time points, indicating that dietary MOS enhanced host antimicrobial ability. In contrast, AKP activity decreased postinfection, and there was no significant difference between the control and MOS200 groups, whereas that of the MOS400 group was lower at 0, 12, and 72 hpi, indicating that only MOS supplementation of 400 mg/kg promoted an inflammatory response upon infection.

Two of the antioxidant enzymes, SOD and GST, exhibited similar tendencies; they were downregulated postinfection, whereas these enzyme activities in the MOS200 and MOS400 groups were drastically higher than those of the control group at most time points. Moreover, CAT activity increased prominently postinfection in the control and MOS200 groups, and that of the MOS supplemental groups was higher than that of the control group at all time points, especially the MOS400 group that maintained activity at a stable higher level. Thus, dietary MOS enhanced the antioxidant ability of juvenile *M. amblycephala* by increasing the activities of SOD, CAT, and GST.

### Dietary MOS Maintain the Stability of Intestinal Histology in Juvenile *M. amblycephala*

**Figure 3:**
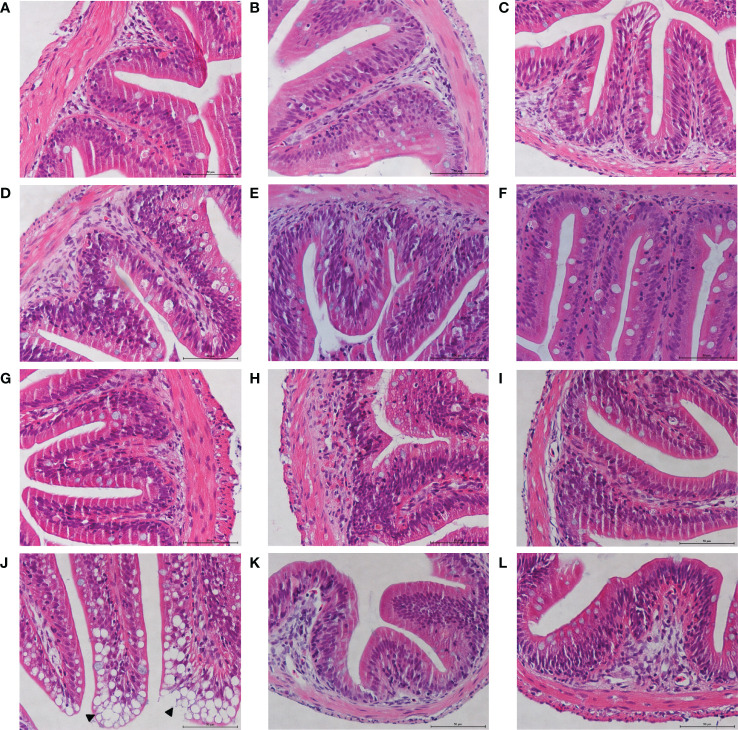
Effects of MOS supplementation on the intestinal histological structures of juvenile *M. amblycephala* by H&E staining. **(A–C)** Mid-intestine sections of control, MOS200, and MOS400 groups at 0 hpi, respectively. **(D–F)** Sections at 12 hpi. **(G–I)** Sections at 24 hpi. **(J–L)** Sections at 72 hpi. The pathological symptoms were marked with triangle. Scale bars represented 50 µm (400×).

**Figure 4:**
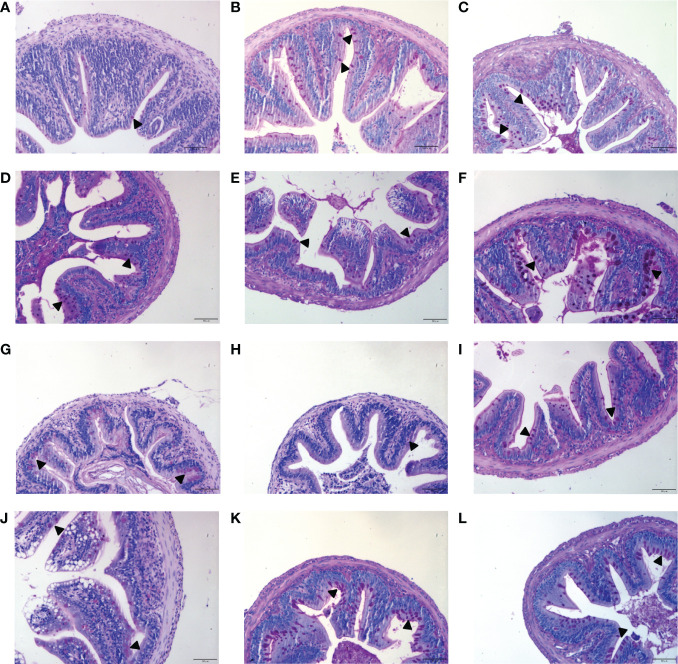
Effects of MOS supplementation on the numbers of intestinal goblet cells by AB-PAS staining. **(A–C)** Mid-intestine sections of control, MOS200, and MOS400 groups at 0 hpi, respectively. **(D–F)** Sections at 12 hpi. **(G–I)** Sections at 24 hpi. **(J–L)** Sections at 72 hpi. Goblet cells were marked with triangle. Scale bars represented 50 µm (200×).

To analyze the effects of MOS on the histological characteristics and number of goblet cells upon infection, juvenile *M. amblycephala* intestinal paraffin sections were prepared for H&E and AB-PAS staining. No significant pathological symptoms were observed in the MOS supplemental groups, whereas typical vacuolization was observed at the end of the intestinal villi of the control group at 72 hpi (**Figure 3**). Combined with the results of the AB-PAS staining, it could be concluded that the vacuolar structures of the control group were not goblet cells but necrosis of the intestinal folds, indicating that MOS supplementation could protect the intestines of *M. amblycephala* from pathological injury. In addition, AB-PAS staining revealed that the number of goblet cells notably increased upon bacterial infection in all groups, and those of the MOS supplemental groups were much greater than those of the control group, indicating that the goblet cells would form a mucosal barrier to protect the epithelial cells (**Figure 4**; **Table 3**).

**Table 3:** Statistical analysis of juvenile *M. amblycephala* intestinal histology fed with or without MOS (mean ± SE).

Items	Groups		
	Control	MOS200	MOS400
Villus length (μm)	133.55 ± 4.13	131.74 ± 3.76	126.75 ± 3.01
Crypt depth (μm)	29.71 ± 1.05	28.47 ± 0.62	30.96 ± 0.61
Microvillus length (μm)	1.04 ± 0.02	1.06 ± 0.03	1.01 ± 0.01
Goblet cells (N/mm^2^): 0 hpi	236.69 ± 47.41^c^	532.21 ± 80.83^b^	732.82 ± 189.55^a^
Goblet cells (N/mm^2^): 12 hpi	675.6 ± 79.35^b^	706.09 ± 140.08^b^	956.14 ± 169.97^a^
Goblet cells (N/mm^2^): 24 hpi	654.68 ± 148.08^b^	473.07 ± 105.45^c^	848.75 ± 183.68^a^
Goblet cells (N/mm^2^): 72 hpi	504.73 ± 125.75^c^	684.32 ± 166.39^b^	1122.24 ± 139.10^a^

In the same row, values with no letter or the same superscript letter meant no significant difference (P > 0.05), while with different superscript letters meant significant differences (P < 0.05).

**Figure 5:**
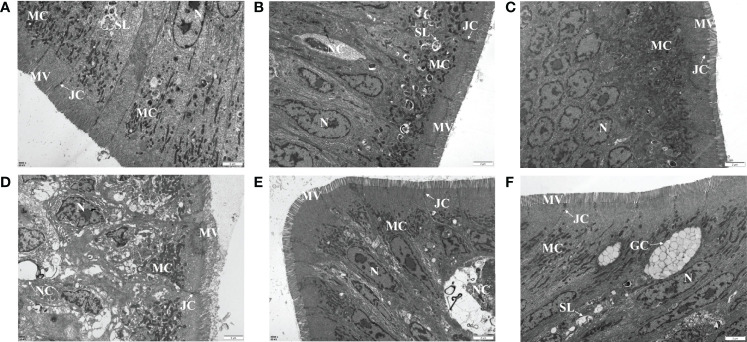
Effects of MOS supplementation on the intestinal ultrastructure of *M. amblycephala* by TEM assay. **(A–C)** Mid-intestine sections of control, MOS200, and MOS400 groups at 0 hpi, respectively. **(D–F)** Sections at 24 hpi. G, goblet cell. Scale bars represented 2 µm (8,000×).

A TEM assay was conducted to estimate the effects of dietary MOS on the ultrastructure of the intestines of juvenile *M. amblycephala*, which showed no notable difference in the intestinal ultrastructure between the MOS supplemental and control groups before infection (**Figures 5A–C**). However, significant disruption of the microvilli and junctional complex was observed in the control group postinfection, which also showed other pathological characteristics, including disorganized histological structures, nuclear atypia, increased pinocytotic vesicles, and partial necrocytosis (**Figure 5D**). In contrast, the intestinal epithelial barriers of the MOS supplemental groups were well-protected upon infection, but necrocytosis was also observed in the MOS200 group (**Figures 5E, F**). Furthermore, goblet cells were found in the MOS400 group, which is consistent with the results of the AB-PAS staining (**Figure 5F**). In addition, the villus length, crypt depth, and microvillus length showed no significant differences among the three groups (**Table 3**), which might have resulted in the undifferentiated growth performance of *M. amblycephala.*

### Dietary MOS Affect the Expression of *M. amblycephala* Intestinal Immune and Tight Junction-Related Genes

**Figure 6:**
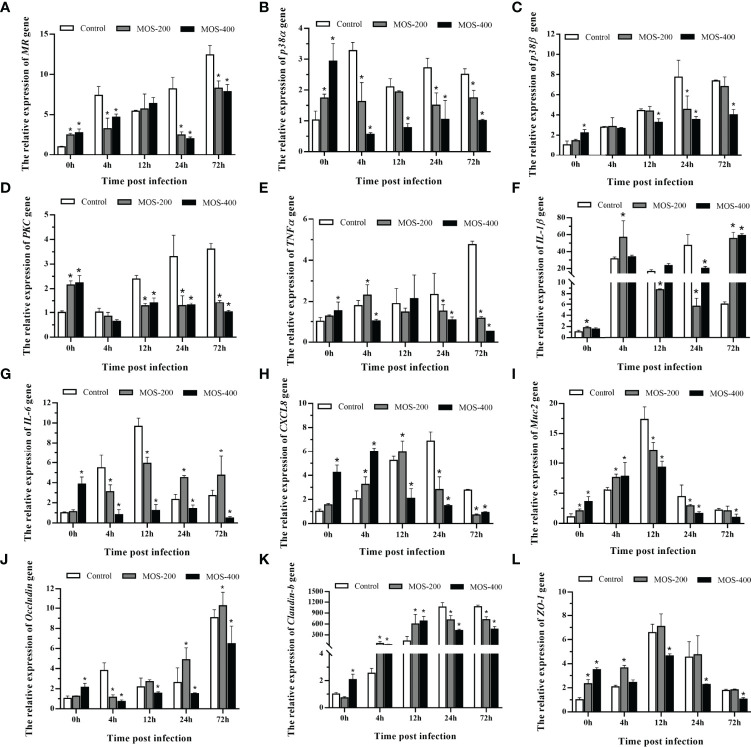
Expression patterns of *M. amblycephala* intestinal immune and tight junction related genes in the three groups upon infection. The detected genes including *MR* **(A)**, *p38α* **(B)**, *p38β* **(C)**, *PKC* **(D)**, *TNFα* **(E)**, *IL-1β* **(F)**, *IL-6* **(G)**, *CXCL8* **(H)**, *Muc2* **(I)**, *Occludin* **(J)**, *Claudin-1* **(K)**, and *ZO-1* **(L)**, and *GAPDH* was selected as the reference gene. Data were shown as mean ± SE, differences were determined by one-way analysis of variance (ANOVA). The asterisks indicated statistically significant differences among different groups at a certain time point (*P* < 0.05).

The expression of *M. amblycephala* intestinal immune and tight junction-related genes was detected in the control and MOS supplemental groups upon infection with *A. hydrophila*. The expression levels of most detected genes were much higher in the MOS supplemental groups after the 8-week feeding experiment, especially in the MOS400 group, indicating that MOS supplementation enhanced the immunity and tight junctions of juvenile *M. amblycephala* (**Figure 6**). In addition, the expression levels of these genes were induced upon infection with *A. hydrophila*, whereas those of the immune genes and related signal factors in the MOS supplemental groups were not increased as significantly as those of the control group postinfection, indicating that MOS supplementation suppressed excessive intestinal inflammation and maintained homeostasis of the host’s physiological functions. Moreover, the expression trend of the *muc2* gene was similar to that of other immune genes, and the MOS supplemental groups maintained a higher expression before 12 hpi, which is consistent with the number of goblet cells detected by histological analysis.

The expression of tight junction-related genes also increased postinfection, especially that of *claudin-b* with a hundredfold upregulation, and the MOS supplemental groups maintained gene expression at stable high levels after 12 hpi. However, the expression levels of the *occludin* and *ZO-1* genes in the MOS400 group were always lower than those in the other groups (**Figure 6**). Combined with the characteristics of intestinal histology, the expression patterns reflected the diverse feedback regulation in the three groups required to maintain the stability of *M. amblycephala* intestinal tight junctions.

### Dietary MOS Improve the Composition of *M. amblycephala* Gut Microbiota

**Figure 7:**
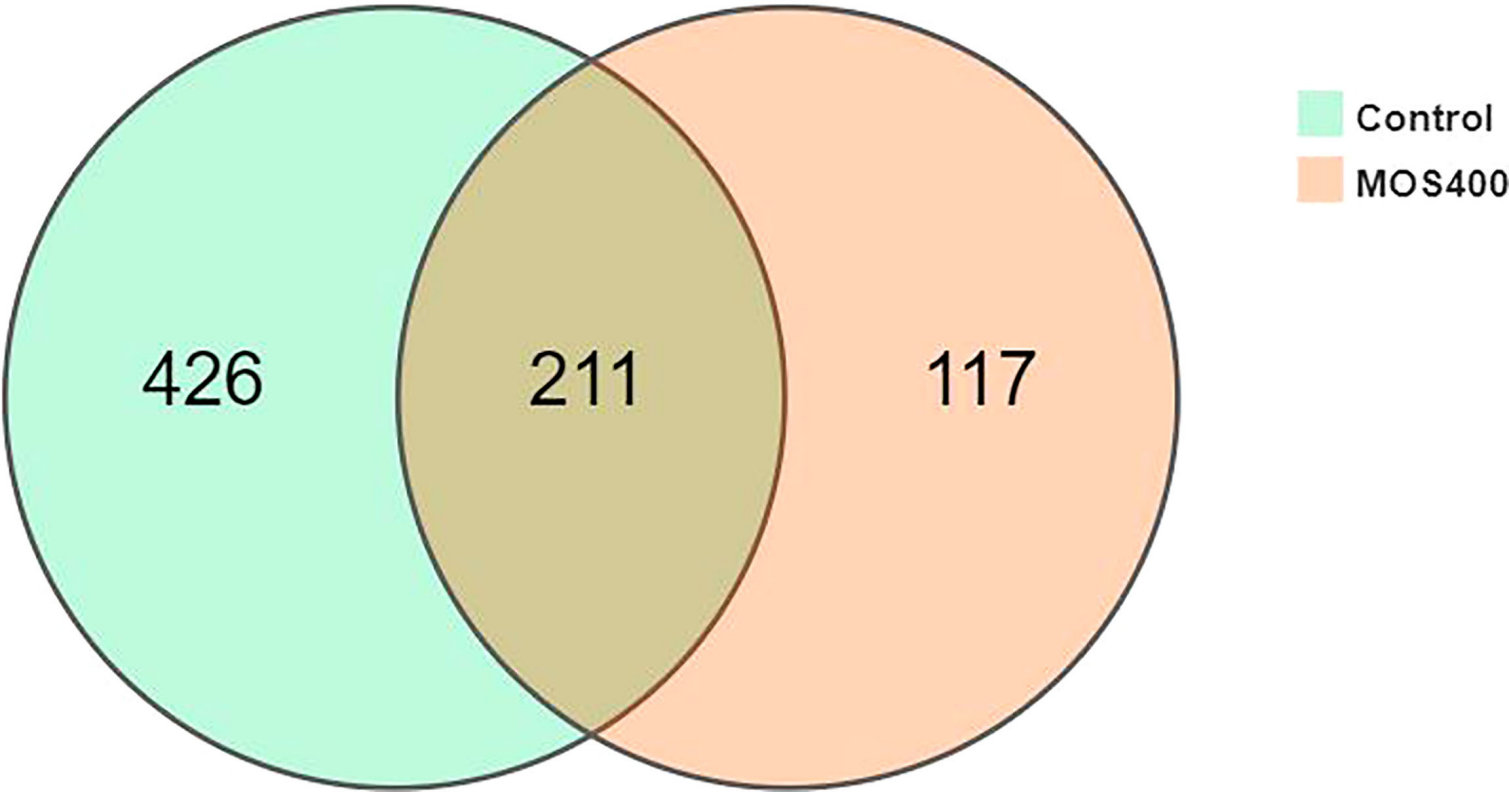
Venn diagram analysis of OTU numbers in the control and MOS400 groups.

A gut microbiome analysis of juvenile *M. amblycephala* was conducted using high-throughput 16S rDNA deep sequencing technology (V3 and V4 regions). The mean obtained clean reads of 14 samples were 134,743 with an average read utilization ratio of 92.59%, and the sequencing coverage was greater than 0.99, which was representative of the samples. As shown in **Figure 7**, dietary MOS decreased the number of specific OTUs of the gut microbiota. Bacterial richness and diversity were analyzed according to the identified OTUs, and the MOS400 group exhibited lower species richness (Chao 1 and Ace) and diversity estimates (Shannon alpha and Simpson) than that in the control group (*P* < 0.05; **Table 4**).

**Table 4:** Richness and diversity statistics of *M. amblycephala* gut microbiota (mean ± SE).

Sample	Chao	Ace	Shannon	Simpson	Coverage
Control	225.43 ± 28.37^a^	225.45 ± 28.38^a^	3.91 ± 0.11^a^	0.05 ± 0.00^b^	1.00 ± 0.00
MOS400	132.00 ± 13.48^b^	132.00 ± 13.48^b^	3.06 ± 0.11^b^	0.11 ± 0.01^a^	1.00 ± 0.00
*P-*value	0.017	0.017	0.001	0.001	0.391

The columns with different letter superscripts were significantly different (P < 0.05).

**Figure 8:**
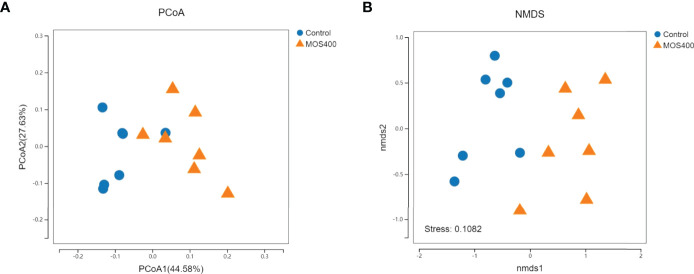
Comparison of gut microbial composition between the control and MOS400 groups with weighted UniFrac PCoA analysis **(A)** and non-metric multidimensional scaling (NMDS) diagram **(B)**.

**Figure 9:**
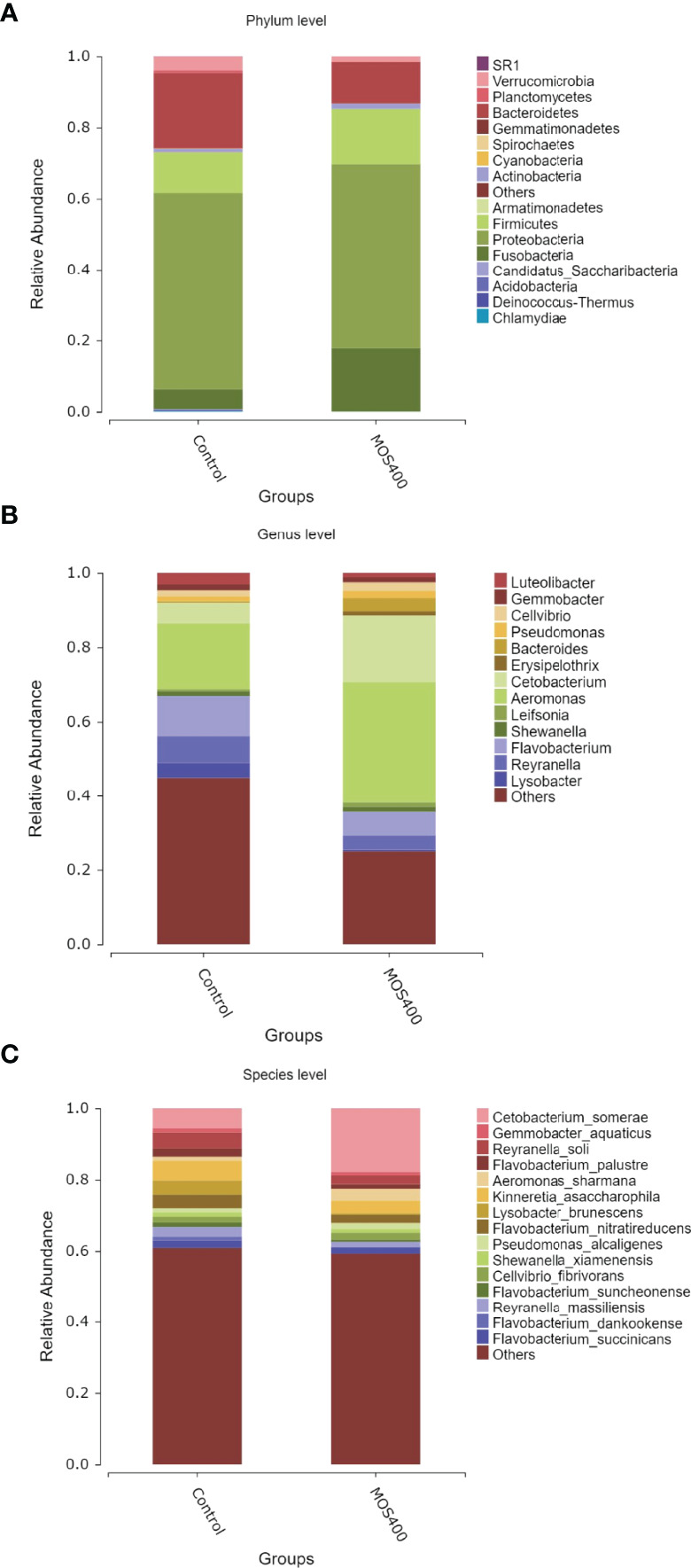
Dietary MOS affected the gut microbial composition of juvenile *M. amblycephala*. Relative abundance of gut microbiota at phylum **(A)**, genus **(B)**, and species **(C)** levels.

Weighted UniFrac PCoA (principal coordinates analysis) and NMDS (non-metric multidimensional scaling) analyses revealed that the replicates of the control and MOS400 groups were not clustered together, indicating that dietary MOS led to differences in the gut microbial composition (**Figure 8**). The gut microbial compositions of juvenile *M. amblycephala* fed with or without dietary MOS at the phylum, genus, and species levels are shown in **Figure 9** and **Supplemental Table 2**. At the phylum level, the highest relative abundance observed in both groups was Proteobacteria, accounting for over 50%. In addition, the relative abundances of Bacteroidetes and Verrucomicrobia were much higher in the control group, whereas Fusobacteria and Firmicutes were more abundant in the MOS400 group (**Figure 9A**). At the genus level, *Aeromonas* was the predominant genus in both groups, and dietary MOS significantly increased the proportion of *Cetobacterium*, but decreased that of *Reyranella* and *Flavobacterium* (**Figure 9B**). At the species level, dietary MOS decreased the relative abundance of dominant bacterial species *Lysobacter brunescens*, *Reyranella soli*, and *Reyranella massiliensis* while upregulated the abundance of *Cetobacterium somerae* and *Aeromonas sharmana*, which became the two most dominant bacterial species in the MOS400 group (**Figure 9C**).

**Figure 10:**
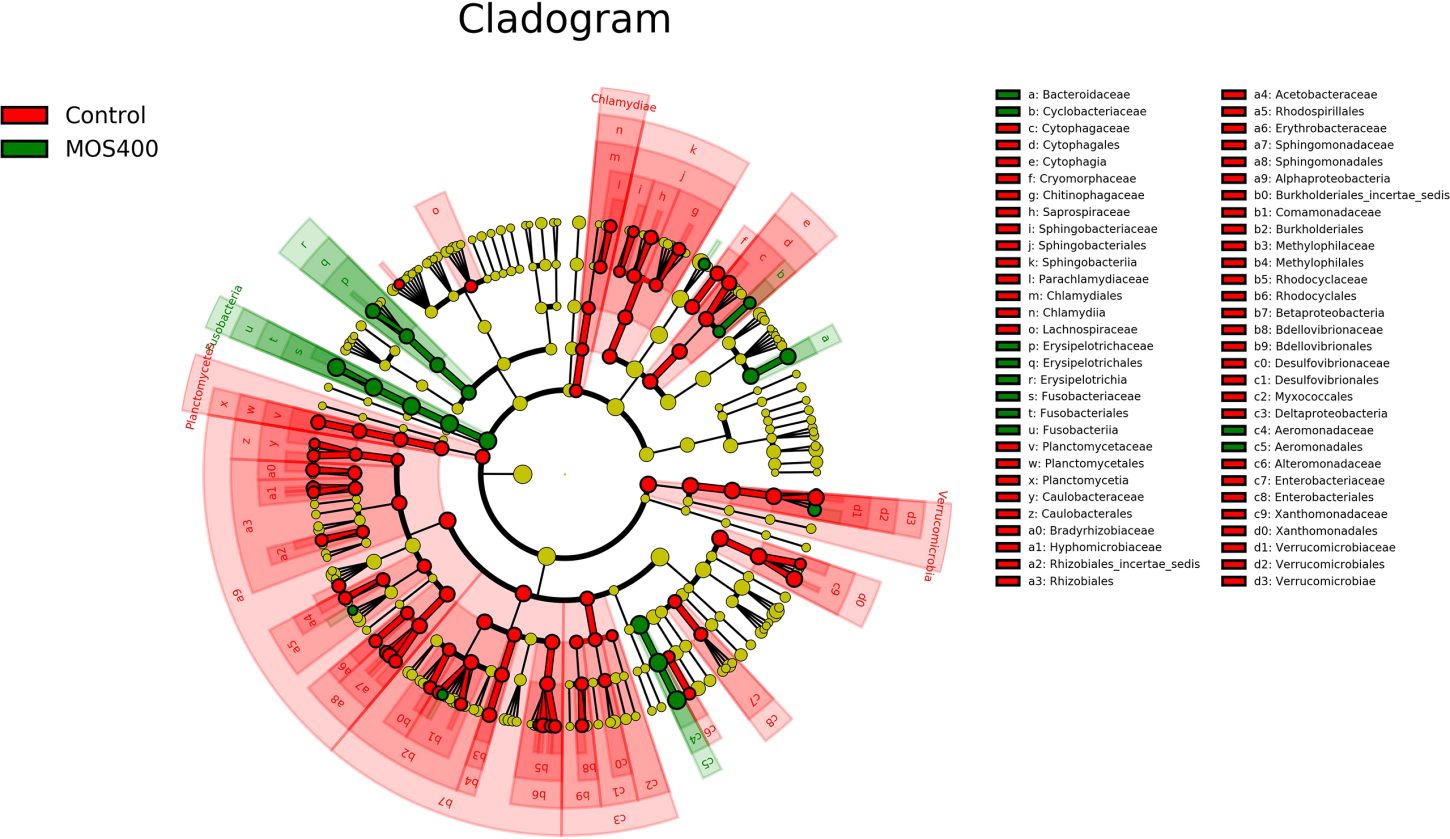
Cladogram revealing the polygenetic distribution of bacterial lineages associated with different groups. Different colors indicated different groups; nodes in red or green represented the microbiome that played important roles in the control or MOS400 groups, whereas yellow nodes indicating the microbiome were not vital in both groups. The circles were in order of phylum, class, order, family, and genus levels from inside to outside.

LDA and LEfSe analysis were conducted to identify possible discriminatory taxa between the two groups at the phylum to genus levels. A total of 106 distinguishing taxa were detected between the control and MOS400 groups, with an LDA score > 3 (**Figure 10**). Specifically, three phyla (Planctomycetes, Verrucomicrobia, and Chlamydiae), eight classes, 17 orders, 25 families, and 33 genera were significantly more abundant in the control group. In comparison, one phylum (Fusobacteria), two classes, three orders, five families, and nine genera were enriched in the MOS400 group.

## Discussion

### Effects of Dietary MOS on the Growth Performance of Aquatic Animals

Previous studies have shown that supplementation with an appropriate amount of MOS can improve feed utilization efficiency and increase the growth of cultured animals. For instance, supplementation with 0.2% MOS could significantly increase the weight gain rate and specific growth rate of juvenile *Oreochromis niloticus* and notably reduce the feed conversion ratio, which has a remarkable promoting effect on the growth of juvenile *O. niloticus* (25). In addition, supplementation with MOS could improve the growth performance of *Ctenopharyngodon idellus*, *Oncorhynchus mykiss*, *Larimichthys crocea*, and *Sparus aurata* (9, 12, 26, 27).

In contrast, the present study found that dietary MOS supplementation had no significant effect on the weight gain rate of juvenile *M. amblycephala* but improved the feed utilization efficiency, which was similar to the findings of the study on *Cyprinus carpio* (28). The reasons for the different effects might be multifactorial, such as the dietary MOS dosage, experimental period, growth stages of the fish, digestive tract characteristics, and gut microbiota composition of different fish species. The digestive tracts of several fish were improved with MOS supplementation, manifesting as an increased length of the intestinal microvilli, villi, or folds, which might promote the absorption and utilization of nutrients and improve growth performance (16, 29, 30). However, the present study found that the villi and microvilli lengths of juvenile *M. amblycephala* were not affected by MOS supplementation, which was consistent with the undifferentiated growth performance of *M. amblycephala*. In addition, we found that dietary MOS increased the abundance of *C. somerae* in the gut microbiota of *M. amblycephala*, which has been shown to improve glucose homeostasis and fish carbohydrate utilization (31), thereby contributing to the improvement of feed utilization efficiency in *M. amblycephala.*

### MOS Decrease the Mortality of Aquatic Animals Upon Pathogenic Infection

The effects of dietary MOS on enhancing disease resistance in aquatic animals have been previously reported. For example, MOS supplementation decreases the cumulative mortality and confinement stress caused by *Vibrio anguillarum* challenge in *Dicentrarchus labrax* (32), similar to the results of studies on *Apostichopus japonicus*, *Carassius auratus gibelio*, *Litopenaeus vannamei*, and *Haliotis discus hannai* Ino (7, 33–35). This indicates that MOS could enhance the resistance of aquatic animals to pathogenic infection. Similarly, the present study also found that cumulative mortality in the short-term MOS supplemental groups decreased significantly, revealing the immunoprotective effects of MOS on juvenile *M. amblycephala.* The possible reasons for this enhanced disease resistance of juvenile *M. amblycephala* might include activation of antioxidases, induced expression of immune genes, maintenance of intestinal histological structures, and improvement of the gut microbial composition (5), which were further verified in the present study.

### MOS Enhance the Activities of Hepatic Antimicrobial and Antioxidant Enzymes in Aquatic Animals

The antimicrobial enzymes, ACP, AKP, and LZM play important roles in host defense and are thus common indicators for evaluating host non-specific immunity (36). LZM can dissolve glycoproteins on the surface of bacteria, and ACP is an enzyme marker for lysosomes with bactericidal effects. Thus, the upregulated activities of ACP and LZM reflected increased host bactericidal effects, which have also been previously observed (7, 35, 37, 38). The activity of AKP was positively correlated with *A. hydrophila* infection levels, and the resistance level of the host to the pathogen was reflected by the significantly lower AKP activity in the immune-stimulated groups (39). We found that AKP activity in the MOS400 group decreased significantly compared with that in other groups upon bacterial infection, indicating that MOS supplementation increased host resistance to bacterial infection (35). Thus, the activities of antimicrobial enzymes revealed the enhanced host non-specific immunity of the MOS supplemental groups, which might result in decreased mortality.

Excess hepatic free radicals produced by stimulation can be scavenged by the antioxidant system, among which SOD, CAT, and GST are important antioxidant enzymes. As the first line of defense in the antioxidant system, SOD can directly capture and dismutate O^2−^ to produce H_2_O_2_, which is further cleared by CAT (40, 41). However, the antioxidant system (particularly SOD activity) is inhibited when the superoxide anion concentration generated in the body is greater than the scavenging capacity of SOD. Thus, decreased activity of SOD is usually observed upon infection, but the present study found much higher SOD activity in the MOS400 group, indicating that dietary MOS could enhance host antioxidant ability (29, 33). Furthermore, CAT activity was more notably induced post-bacterial infection in the MOS supplemental groups, thereby protecting cells from oxidative damage. GST is a key enzyme catalyzing the initial step in the glutathione binding reaction, and its activity was also inhibited postinfection, whereas dietary MOS could maintain its activity in the present study. Similar results have been reported in grass carp (38). In summary, dietary MOS enhanced the antioxidant ability of juvenile *M. amblycephala* by inducing or maintaining the activities of SOD, CAT, and GST, which could assist in increasing the survival rate of *M. amblycephala* upon infection.

### The Effects of Dietary MOS on the Intestinal Histology of Aquatic Animals

Studies have shown that dietary MOS can increase the intestinal villus length and muscle layer thickness of *Anguilla japonica* (30). Similarly, dietary MOS also increase the villus length in the soybean meal group in European sea bass but had no significant effect on the villus width (16). In addition, MOS supplementation had a significant effect on the villus length of juvenile *Pangasianodon hypophthalmus* but did not affect the villus width and crypt depth (42). At the ultrastructural level, TEM assays demonstrated that MOS supplementation could significantly increase the intestinal microvilli length in juvenile Pacific white shrimp (29) and the intestinal microvilli density and length in *Sparus aurata* (12).

In contrast, dietary MOS showed no significant influence on the intestinal villus width and length in Gulf sturgeon (*Acipenser oxyrinchus desotoi*) (43). Similarly, the present study found that dietary MOS had no significant effect on the lengths of the intestinal villi and microvilli of juvenile *M. amblycephala*. The different effects of dietary MOS on fish intestinal histological structures might be related to the supplemental amount, species, and growth stages of the experimental fish. The lengths of intestinal villi and microvilli mainly affect the absorption of nutrients. Thus, dietary MOS exhibited no significant effect on the intestinal villi and microvilli length, resulting in undifferentiated growth performance in *M. amblycephala*. However, dietary MOS could assist in maintaining the stability of the intestinal histological structures and increasing the number of goblet cells upon infection, which might protect the intestinal epithelial barrier in *M. amblycephala*, thereby contributing to improved host immune defense ability and decreased cumulative mortality.

### The Effects of MOS on the Expression of Immune-Related Genes in Various Aquatic Animals

The effects of dietary MOS on fish immunity and other biological functions could be relevant in activating or inhibiting related signaling pathways, reflected as the expression of pathway genes. In *C. idella*, the expression of antioxidant-, apoptosis-, tight junction-, and immune-related genes was regulated by dietary MOS, among which the expression of most antioxidant and tight junction-related genes was induced, whereas that of pro-apoptotic and pro-inflammatory factors was reduced (38). Similarly, the expression of antioxidant-related genes was also upregulated in the intestines (9). Moreover, immune parameters, including antibacterial and anti-inflammatory cytokines, were activated in the spleen and kidneys by MOS supplementation, whereas pro-inflammatory cytokine levels were inhibited (37).

However, the regulatory effects of dietary MOS on the expression of immune-related genes exhibit variable patterns in different species. For instance, the expression of pro-inflammatory cytokines was induced by MOS supplementation in Pacific white shrimp and *O. niloticus* (26, 35). Similarly, in the present study, the expression of *M. amblycephala* pro-inflammatory cytokines, tight junction-related genes, and signaling factors was also increased after 8 weeks of feeding with dietary MOS, indicating that MOS could enhance the intestinal immunity and tight junctions of *M. amblycephala.* However, the expression of pro-inflammatory cytokines in the MOS supplemental groups was not upregulated as significantly as that in the control group upon bacterial infection, revealing that MOS supplementation reduced the damage caused by an excessive intestinal inflammatory response. In addition, the expression of tight junction-related genes also showed different patterns in the control and MOS supplemental groups, which could indicate different feedback regulation of the stability of the junctional complex, as the ultrastructures of the control group were partially disordered upon infection. Therefore, the gene expression patterns indicated that dietary MOS not only possessed immune-enhancing properties but could also prevent excessive inflammation postinfection, thereby decreasing the mortality caused by *A. hydrophila* infection.

### The Dietary MOS Regulate the Richness and Composition of the Gut Microbiota

The gut microbiota of many teleosts is composed of a high abundance of Proteobacteria, Fusobacteria, and Firmicutes (44–47). Proteobacteria was found to be the predominant phylum in the present study. The effect of MOS on the gut microbiota lacks consensus (5, 12, 32, 48, 49). The addition of dietary MOS may improve the gut microbial community by increasing the abundance of beneficial bacteria, thus enhancing host disease resistance, feed utilization, and growth performance. The present study found that dietary MOS supplementation affected the gut microbial diversity and composition, especially the abundance of Verrucomicrobia, Bacteroidetes, Fusobacteria, and Firmicutes, which could contribute to the improvement of feed utilization and anti-infection ability.

In addition, dietary MOS increased the abundance of *Aeromonas* and *Cetobacterium* in the intestines of juvenile *M. amblycephala*, which were the dominant genera in the fish intestines. Previously, *Cetobacterium* was isolated from several fish intestines, which mainly consisted of *C. somerae*. Recently, *C. somerae* has been developed as an aquatic probiotic strain with lipid-lowering, anti-inflammatory, anti-apoptotic, and antiviral functions. This species has also been reported to play a role in regulating *Danio rerio* glucose homeostasis (31), and its fermentation product could improve the gut health of *C. carpio* and *D. rerio* (50, 51). Thus, it can be speculated that dietary MOS improved the gut health of juvenile *M. amblycephala* by increasing the relative abundance of beneficial bacteria.

## Conclusions

In conclusion, this study revealed that dietary MOS improved feed utilization efficiency, intestinal health, and resistance to infection in juvenile *M. amblycephala* and can therefore be used as a functional feed additive and immunostimulant. Most importantly, MOS supplementation promoted intestinal health by maintaining intestinal homeostasis and the balance between enhancing anti-infection immunity and preventing excessive inflammation with significant immune-protective effects in juvenile *M. amblycephala*.

## Data Availability Statement

The datasets presented in this study can be found in online repositories. The names of the repository/repositories and accession number(s) can be found below: https://www.ncbi.nlm.nih.gov/search/all/?term=PRJNA804329.

## Ethics Statement

The animal study was reviewed and approved by Animal Care and Use Committee of Jiangsu Ocean University (protocol no. 2020-37, approval date: September 1, 2019).

## Author Contributions

Conceptualization: ZD and HC; methodology: YZ; software: HL; validation: JX; formal analysis: MZ; investigation: XW, YL, and XZ; resources: XC; data curation: YH; writing—original draft preparation: ZD and XW; writing—review and editing: ZD and XZ; visualization: XW; supervision: ZD and XZ; project administration: XZ; funding acquisition: ZD and HC. All authors contributed to the article and approved the submitted version.

## Funding

This work was supported by Chinese Postdoctoral Science Foundation (grant number 2020M671386); the Natural Science Foundation of Jiangsu Province, China (grant numbers BK20181071 and BK20201465); Postdoctoral Science Foundation of Jiangsu Province (grant number 2020Z157) and Lianyungang City (China); Postgraduate Research & Practice Innovation Program of Jiangsu Province (grant number KYCX2021-019); and the Priority Academic Program Development of Jiangsu Higher Education Institutions. 

## Conflict of Interest

The authors declare that the research was conducted in the absence of any commercial or financial relationships that could be construed as a potential conflict of interest.

## Publisher’s Note

All claims expressed in this article are solely those of the authors and do not necessarily represent those of their affiliated organizations, or those of the publisher, the editors and the reviewers. Any product that may be evaluated in this article, or claim that may be made by its manufacturer, is not guaranteed or endorsed by the publisher.

## Abbreviations

MOS, mannan oligosaccharides; MR, mannose receptor; CFU, colony forming unit; LD50, median lethal dose; muc2, mucin 2; RTgutGC, Oncorhynchus mykiss intestinal epithelial cell line; MS-222, 3-aminobenzoic acid ethyl ester methane sulfonate; ACP, acid phosphatase; AKP, alkaline phosphatase; CAT, catalase; SOD, superoxide dismutase; GST, glutathione S-transferase; LZM, lysozyme; H&E, Hematoxylin and eosin staining; AB-PAS, Alcian blue and periodic acid Schiff staining; TEM, transmission electron microscopy; qRT-PCR, real-time quantitative reverse transcription polymerase chain reaction; GAPDH, glyceraldehyde-3-phosphate dehydrogenase; FLASH, Fast Length Adjustment of Short reads; OTUs, operational taxonomic units; DADA2, Divisive Amplicon Denoising Algorithm; PCoA, principal coordinates analysis; LDA, linear discriminant analysis; LEfSe, LDA effect size; NMDS, non-metric multidimensional scaling.
