# Supplementary material for: Dietary Mannan Oligosaccharides Enhance the Non-Specific Immunity, Intestinal Health, and Resistance Capacity of Juvenile Blunt Snout Bream (Megalobrama amblycephala) Against Aeromonas hydrophila
Source: Front Immunol. 2022 Jun 15;13:863657. doi: 10.3389/fimmu.2022.863657 (PMC9240629; doi:10.3389/fimmu.2022.863657)
Supplement: Supplementary file 1 [file DataSheet_1.docx]

**Supplemental Table 1.** Primers used for qRT-PCR in the present study.

| **Primers** | **Sequences (5’-3’)** |
| --- | --- |
| q*MR*-F | GCCATTCTGTTCCTACCATTCC |
| q*MR*-R | CCTTCCCGCAGACATACATCTC |
| q*PKC*-F | GTAAATCCACCAAGCGACCTG |
| q*PKC*-R | CCCTCCCATAGACGACCATAAG |
| q*p38α*-F | CTTCCCCGTGTTCTTATCCA |
| q*p38α*-R | ACATTCTCTGTGTCCTTTTGG |
| q*p38β*-F | TTTTTGTATTTGATGTGAGGC |
| q*p38β*-R | CTCCTCCTCTTGCTTGGTTTA |
| q*TNFα*-F | CCGCTGCTGTCTGCTTCA |
| q*TNFα*-R | GCCTGGTCCTGGTTCACTCT |
| q*IL1β*-F | GTGCCAGGTGCCAAGTAGC |
| q*L1β*-R | AAGCCCAAGATATGCAGGAGT |
| q*IL6*-F | ACAGCAGTATGGGGGAGTTAT |
| q*IL6*-R | TTCATCACGCAGAGTTTTCAC |
| q*iNOS*-F | ATTCAAGGGCAGCTTCCAGG |
| q*iNOS*-R | CAGGGGCAAAGTTTAAGGGC |
| q*CXCL8*-F | TATTGTTGCTGTGGCATTTGTG |
| q*CXCL8*-R | TGGTTTCCTTCAGGGTGGC |
| q*Muc2*-F | CTGCCAAAGCCTCATTCAC |
| q*Muc2*-R | TGATACTAACTGACACCCTGCTGA |
| q*occludin*-F | TATCTGTATCACTACTGCGTCG |
| q*occludin*-R | CATTCACCCAATCCTCCA |
| q*claudin*-F | GAGGGAATCTGGATGAGC |
| q*claudin*-R | ATGGCAATGATGGTGAGA |
| q*ZO-1*-F | CGGTGTCTTCGTAGTCGG |
| q*ZO-1*-R | CAGTTGGTTTGGGTTTCAG |
| q*β-actin*-F | GCTCTTACAGGAAACGGGTC |
| q*β-actin*-R | GCAGCAGCTCTGTAGGTCAT |
| q*EF1α*-F | CTTCTCAGGCTGACTGTGC |
| q*EF1α*-R | CCGCTAGCATTACCCTCC |
| q*GAPDH*-F | TGCCGGCATCTCCCTCAA |
| q*GAPDH*-R | TCAGCAACACGGTGGCTGTAG |

**Supplemental Table 2.** Relative abundance of gut microbiota of juvenile *M. amblycephala* fed with different diets at phylum-genus-species levels (%).

| **Taxonomic level** | **Gut microbiota** | **Groups** | |
| --- | --- | --- | --- |
|  |  | **Control** | **MOS400** |
| Phylum | Proteobacteria | 55.3 | 51.8 |
|  | Bacteroidetes | 21.2 | 11.6 |
|  | Firmicutes | 11.4 | 15.5 |
|  | Fusobacteria | 5.6 | 17.8 |
|  | Verrucomicrobia | 3.9 | 1.5 |
|  | Actinobacteria | 0.8 | 1.5 |
| Genus | *Aeromonas* | 17.6 | 32.6 |
|  | *Cetobacterium* | 5.6 | 17.8 |
|  | *Flavobacterium* | 10.8 | 6.4 |
|  | *Reyranella* | 7.2 | 3.8 |
|  | *Lysobacter* | 4.0 | 0.4 |
|  | *Luteolibacter* | 3.1 | 1.2 |
|  | *Bacteroides* | 0.4 | 3.5 |
| Species | *Cetobacterium somerae* | 5.6 | 17.8 |
|  | *Aeromonas sharmana* | 1.1 | 3.4 |
|  | *Kinneretia asaccharophila* | 5.5 | 3.5 |
|  | *Reyranella soli* | 4.5 | 2.5 |
|  | *Lysobacter brunescens* | 4.0 | 0.4 |
|  | *Flavobacterium nitratireducens* | 3.8 | 2.3 |
|  | *Reyranella massiliensis* | 2.7 | 1.3 |
|  | *Flavobacterium palustre* | 2.3 | 1.2 |
